# Assessing Condom Use and Views on HIV Counselling and Testing among TVET College Students in Limpopo Province, South Africa

**DOI:** 10.3390/ijerph20116044

**Published:** 2023-06-03

**Authors:** Mimi Eve Teffo, Mathildah Mpata Mokgatle

**Affiliations:** Department of Epidemiology and Biostatistics, School of Public Health, Sefako Makgatho Health Sciences University, Pretoria Ga-Rankuwa 0208, South Africa; mathildah.mokgatle@smu.ac.za

**Keywords:** condom use, HIV, HCT, sexual behaviour, young people, college students, South Africa

## Abstract

South Africa’s population is predominantly young, presenting a powerful resource for the country. Despite this, adolescents and young people remain at the epicentre of the HIV epidemic, particularly adolescent girls and young women (AGYW). There are limited studies that investigate the views on HIV Counselling and Testing (HCT) and condom use among adolescents and young people, and college students in particular, in South Africa. This cross-sectional study assessed condom use among college students and their views and opinions on HCT. Utilising an adapted questionnaire from the Australian Secondary students and the South African Sexual Health survey, the data from 396 students were analysed using univariate and multiple logistic regression performed using Stata IC version 16. The majority of the students (*n* = 339, 85.8%) had a sexual partner at the time of the study. Our findings reveal a relatively high occurrence of condom use in the last sexual encounter (*n* = 225, 60%) and high HCT uptake (*n* = 50, 88.4%). Females were generally more comfortable regarding HIV services compared to their male counterparts. More than half, 54.6% vs. 36.0% were comfortable about testing, 34.0% vs. 48.3% felt very scared about testing for HIV, 3.6% vs. 10.1% reported that they were not ready to take an HIV test, 7.6% vs. 5.6% intended getting tested soon (*p* = 0.0002). Condom use was significantly associated with the use of a condom during the first sexual encounter (aOR = 4.71, 95% CI: 2.14, 10.37) and knowing their partner’s HIV status (aOR = 2.08, 95% CI: 1.19–3.65). The HCT and condom promotion strategies implemented by Higher Health in TVET colleges is showing success and colleges in other parts of the region could emulate these best practices. Program developers should consider tailor-made combinations of prevention interventions that would appeal to both female and male college students to improve condom use and uptake of HIV testing services.

## 1. Introduction

Africa has an increasingly young population compared to the rest of the world, with 70% of sub-Saharan Africa residents below the age of 30, providing an opportunity for the continent’s growth and sustainable development [1]. Similar to the other countries in the SADC region, South Africa’s (SA) young people constituted almost a third of the population (17.84 million) in 2019 according to mid-year estimates [2]. Although this is a great asset for the country, young people in SA continue to be vulnerable to the HIV pandemic. The results from the fifth national population-based survey showed that 38% of new HIV infections were found among youth aged 15–24 years. In fact, females in this age category accounted for the majority of new HIV infections among youths with the highest burden of 1.51% [3]. The committed high level leadership in SA recently stimulated political buy-in and collective commitment from diverse stakeholders to address HIV particularly among adolescent girls and young women [4]. For example, HIV testing services are offered to students in college campuses and condoms are easily accessible and offered freely at various campus points such as campus residences, campus clinics and during First Things First Campaign, a campaign developed to mitigate the spread of HIV and STIs. It is worth noting is that the provision of flavoured condoms is a strategy that is used to make condoms appealing to students. Additionally, HIV testing campaigns are complemented by activities that help facilitate the distribution and effective use of condoms [5]. Moreover, the SA government has made huge strides in HIV responses for young people such as implementation of a large-scale prevention program such as the United States of America (U.S.) President’s Emergency Plan for AIDS Relief (PEPFAR) DREAMS (Determined, Resilient, Empowered, AIDS-Free, Mentored and Safe) initiative which exists in 10 countries in Sub-Saharan Africa [6]. This program aims to address structural and behavioural drivers that increase the risk of acquiring HIV and gender-based violence among adolescent girls and young women (AGYW) through the implementation of evidence-based interventions. The Global Fund also provides funding to support the Adolescents and Young People (AYP) Program in the country for the implementation of HIV interventions targeted at in and out of school AYP including Technical Vocational Education and Training (TVET) colleges through Higher Health (formerly HEAIDS).

The Joint United Nations Program on HIV/AIDS has outlined a new set of ambitious targets that aims for HIV testing, treatment and suppression rates to be 95-95-95 by 2025 [7]. Despite SA’s commitment to these targets and SA’s robust national strategic plan on HIV, Tuberculosis (TB) and Sexually Transmitted Infections (STIs) 2017–2022, HIV remains a significant public health threat particularly amongst females aged 15–24 years. Seemingly, as in other regions of Sub-Saharan Africa, students in institutions of higher learning fall within the similar age category, and as such, they are classified as a group vulnerable to contracting HIV [8,9].

A growing body of evidence shows that young people engage in risky sexual behaviour such as inconsistent condom use, and concurrent sexual partnerships [8,10,11,12,13]. Furthermore, AGYW enter into sexual partnerships with older male counterparts and evidence indicates that these age-disparate relationships make the AGYW more vulnerable since older men are most likely to be HIV positive [14,15,16].

Previous studies suggest that condom use as a primary prevention measure among college and university students remains low. A pilot survey among university students at a rural SA university reported that most participants (56.6%) engaged in condomless sex during their first sexual intercourse [17]. Additionally, a study among students studying in six different universities in Sudan showed that the majority of the students who were sexually active reported that most of their sexual practices were unprotected [18]. Compared to one previously conducted study, we found that less than half (40%) of the sexually active students did not use a condom in their last sexual encounter [19]. These results correlate (48%) with those of a DRC study among university students in Kikwit University [20] although they are lower than the findings (66.5%) of a study conducted among TVET students in SA [21]. 

Moreover, HIV testing remains the most critical intervention to HIV prevention and entry into continuum of care for those who test positive. Although previous studies argue that higher education is protective, and students are more likely to have been tested for HIV [22,23], this is in contrast to similar studies conducted in the region were the majority of students reported that they were sexually active and most of their sexual encounters were unprotected [18,19,24,25]. Additionally, previous studies conducted revealed that university students engage in sexual encounters while intoxicated [22,26] and often do not take precautionary measures to reduce HIV infections such as using condoms correctly and consistently [27,28,29]. 

Despite the presence of HIV interventions at TVET colleges through Higher Health in SA, there is a scant literature on the views of HIV Counselling and Testing (HCT) and condom use among college students as the end-users of these services. We employed a descriptive study design to assess condom use and HCT views among TVET students to address these gaps in knowledge.

## 2. Materials and Methods

### 2.1. Study Design and Population

This cross-sectional study was conducted in Capricorn College on the TVET Polokwane Campus, the second largest college in Polokwane Municipality, Limpopo Province, South Africa. At the time the study was carried out, approximately 5600 students from different parts of the province were registered at the college. The students are distributed across Level 2 to Level 4 of study in vocational, occupational and artisan skills and education. We recruited *n* = 396 students to participate in the study.

### 2.2. Sample and Sampling Technique

To allow for a variety of students’ participation by qualification type, students from all programs were included in the study. The programs were selected by simple random sampling. The classes were randomly selected from National Qualifications Framework (NQF) Level 2 to NQF Level 4, and the researcher conveniently took everybody in the class that volunteered to participate in the study. The sample size was determined using the statistical method Raosoft Calculator at a confidence interval of 95% and 5% margin of error. The target population size was 5600, the response distribution was 50% and the sample size for the study was 360 participants. The researcher raised the sample size by 10% to cater for unusable and incomplete questionnaires as they were self-administered, and the ultimate total sample was 396 participants.

### 2.3. Measures

We used a validated structured questionnaire from an Australian Secondary students and South African Sexual Health Survey [21,30]. The study assessed the participants’ socio-demographic backgrounds, sexual behaviour, and practice of and opinions on HCT. To assess the risky sexual behaviours of students, we compiled questions in a five-item measure. We asked students if they have a sexual partner, whether they were sexually active, number of sexual partners in the past year, and whether they used a condom during the first and last sexual encounter. The responses were categorized as “yes” and “no” and for number of sexual partners, responses were categorized as “one partner”, “two partners” or “more than two partners”. A 17-item measure was used to assess the practices and opinions on HCT. The questions on having been tested for HIV, adequate information about HCT, intention to test in the future, and comfort with using HCT services were assessed with a “yes”, “no” and “unsure” responses.

### 2.4. Data Collection

All data were collected by means of a self-administered questionnaire. A meeting was arranged with the college management and head of the department to explain the purpose of the study. The course facilitators facilitated the selection of classes, followed by random selection of classes. The students were invited to participate and were further informed that participation in the study was voluntary. We also informed students orally beforehand about the purpose of the study. The questionnaire was administered by the first author (MET) and the questionnaire was administered in English between 8 h 30 and 13 h 30 each day during free classes to avoid curriculum interruptions.

### 2.5. Data Analysis

After data collection, the questionnaires were coded and all data were entered into Excel. The data were analysed using STATA IC Version 16. The collected data were summarized using frequencies, percentages and odds ratios. Chi-squared tests were performed in order to assess whether there was an association between condom use as an outcome and the other independent variables and level of significance was determined. Moreover, logistic regression was used, and only statistically significant variables were included from the bivariate analysis. The unadjusted odds ratios (ORs) that were found during the bivariate analysis at a confidence level of 95% and *p* < 0.05 level of statistical significance were used to compute the multiple logistic regression model using a backward method. The factors associated with condom use were presented as adjusted ORs and statistical significance was set as *p*-value < 0.05.

### 2.6. Ethical Considerations

Ethical clearance for the study was obtained from the Research and Ethics Committee of Sefako Makgatho Health Sciences University (SMUREC/H/214/2017: PG). We further received permission from Capricorn College for the TVET Central Office and the college management. Each student signed an informed consent form prior to participation in the study. Moreover, students were reassured that they could withdraw from the study at any time. The students were reassured of the confidentiality and anonymity of their responses. To maintain this, we did not capture personal identifiers such as names and addresses, and only the age and gender of the students were linked to the questionnaires.

## 3. Results

### 3.1. Sociodemographic, Sexual Behaviour and Condom Usage of the College Students

All students that were recruited (*n* = 396) completed the questionnaire giving a response rate of 100%. The mean (SD) age of the students was 22.9 years old (SD: 2.7). The students were predominantly female (77.2%). Almost half (45.5%) of the students were studying at Level 3 and the majority (74.9%) were residing in rural areas. The majority (85.8%) had a sexual partner at the time of the study. Of the sexually active students, 63.7% had one partner, 18.7% had two partners and 17.6% had more than two partners. With respect to condom usage, most (87.4%) students used a condom the first time they had sex and only 60% of the students used a condom the last time they had a sexual encounter. The students’ sociodemographic, sexual behaviours and condom usage characteristics are shown in Table 1.

### 3.2. Testing Practices of the College Students

Of all the students, 88.4% reported that they have been tested for HIV before. Meanwhile, 11.6% reported to have never been tested for HIV. On knowing the HIV status of their sexual partner, the results showed that more than half (60.4%) knew the HIV status of their current sexual partner. The data further show that 76.0% of the students were likely to discuss HIV testing with their partner. Moreover, an overwhelming 81.0% of the students expressed that they were likely to ask their partner to go for HIV testing, 12.6% were unlikely to ask their partner while 6.4% would never ask their partner to go for HIV testing (see Table 2 cited in https://hsag.co.za/index.php/hsag/article/view/2095, accessed on 25 May 2023). 

### 3.3. Reasons for Testing and Non-Uptake of HCT

Of the students that have been tested for HCT, 30 (8.6%) were tested at the hospital, 265 (76.2%) at a clinic, 12 (3.5%) at a private doctor, 33 (9.5%) during an HIV testing campaign and 8 (2.3%) at other service points. The reason for undergoing HIV testing for the majority of the students (279; 79.9%) was to know their status; other reasons included being pregnant (22; 6.3%), being sexually active (9; 2.6%), tested with a partner (14; 4.0%), or were offered a test by a healthcare provider (25; 7.2%). Moreover, the majority of the students (333; 96.2%) expressed satisfaction with the HCT services. Meanwhile, the students had varied reasons for non-uptake of HCT: 29.6% felt that they were not at risk of contracting HIV, 20.5% were not being sexually active, 9.1% were unsure if the results will be kept confidential and 6.8% feared the stigma associated with a positive result.

### 3.4. Views on HIV Counselling and Testing by Gender

Table 3 below summarises the results on HCT views by gender. Relative to the male students, more females expressed that they were comfortable taking an HIV test (54.6% compared to 36.0%), and the difference was statistically significant (*p* = 0.002). There were no statistically significant differences in the proportions of female and male students on the importance of HCT for young people (*p* = 0.854), the place for HCT as a motivation for a person to use HTS (*p* = 0.188), adequate information about HIV services in the facility (*p* = 0.369), and the worry about getting infected with HIV (*p* = 0.701).

### 3.5. Factors Associated with Condom Use

Table 4 shows the results from the logistic regression. The independent variables included gender, level of study, being sexually active, use of condom the first time, had been tested for HIV, know HIV status of partner, likely to discuss HIV testing with partner, and likely to ask partner to go for HIV testing. We started by analysing the computed bivariate analysis and then used multiple logistic models to confirm the association. The bivariate logistic regression showed that seven variables were found to have a *p*-value of <0.05. These were gender, being sexually active, use of condom the first time, had been tested for HIV, knowing HIV status of partner, discussing HIV status with partner and asking partner to go for HIV testing. On multivariate logistic regression, the aOR revealed that using condoms the first time and knowing the HIV status of partner are associated with condom use [OR = 4.71 (95% CI: 2.14–10.37, *p*-value 0.000] and [OR = 2.08 (95% CI: 1.19–3.65, *p*-value 0.000], respectively.

## 4. Discussion

This study assessed condom use and views on HCT in a population of TVET students in Limpopo Province, South Africa. We found that the prevalence of condom use the first time they had sex among students was 87.4%. This was slightly lower than the rate during the last sexual encounter, with 60% of students reporting that they used condoms. Our findings, along with others, show a relatively high condom use in the last sexual encounter [20,21]. This shows that the setting and interventions, such as the First Things First campaign, may have played a role towards condom uptake [5]. The results are consistent with those of studies conducted in Europe, Canada and Brazil with a prevalence of above 40% [31,32]. The study among university students in sub-Saharan Africa reported that 50.5% of the students used a condom during their last sexual intercourse [33]. Additionally, the study among sexually active university students in Mozambique revealed that 74% of the study participants used condoms during their last sexual encounter [25]. Another study showed that 80% of students in a Ugandan university used a condom in their last sex act [34]. A potential reason for the high condom use found in this study may be due to the selection of the study population which was only female. It is worth noting that 55% of students in South African universities reported condom use during their last sexual encounter with steady partners [9]. Moreover, our study identified two significant factors associated with condom use that may inform behavioural and structural interventions aimed at promoting condom use among students in institutions of higher learning.

First and foremost, we found that condom use during the first sexual encounter is associated with condom use. These results correlate with previous studies in Malaysia, Brazil and northern Thailand [32,35,36]. It is critical to note that our present study did not assess whether the college students engaged in the last sexual encounter with a steady partner. Nonetheless, this shows that students are actively considering using condoms and are adequately prepared before they can even engage in their first sexual intercourse [35]. Furthermore, knowledge of partner’s status was found to be associated with condom use. In this study, college students that are aware of their partner’s status were more likely to use condoms. This finding might be due to Higher Health HIV prevention interventions on college campuses, which promote couple testing and disclosure and the importance of using condoms correctly and consistently. [35] also concurred that the longer young people receive health promotion interventions, the better they will translate that knowledge into practice, mainly through consistent condom use. However, we found no statistically significant association between being sexually active and condom use.

We further found that an overwhelming 88.4% of the students have been previously tested for HIV. Compared with a study conducted in a similar setting in TVET colleges in a rural and urban province in South Africa, 72% of students indicated that they have been previously tested for HIV which is comparable to our findings [21]. This result is higher than that found by studies conducted among university students in other countries [25,27,37]. Additionally, our quantitative results show that there are differences in perceived use of HCT services between females and males. Whilst 26.0% of the male students expressed that it is very easy for young people to access HCT, 27.0% of the female students also expressed that it is very easy to access HCT.

By its very nature, HCT remains the key biomedical strategy and a gateway to prevention, care and support, particularly among adolescents and young people. We found that 79.9% of the students in our study reported that they tested to know their HIV status. This result matches those of [21] in South Africa. The available literature has shown a link between increased uptake of HCT and reduced risk of HIV infections [38,39]. Over three quarters of the students reported that HCT is important for young people. These findings corroborate the results of other studies conducted among students in the Democratic Republic of Congo and South African TVET colleges [20,21]. Our results also indicate that students who have not been tested for HIV cited various reasons such as fear of stigma, not being sure if the results will be kept confidential, not wanting to know one’s HIV status, not being sexually active and not at risk of contracting HIV. Our finding is consistent with that of previous studies [21,40].

Eighty-one percent of the students in this study reported that they were sexually active and 18% reported that they have had more than two sexual partners in the past year. Previous studies demonstrated mixed findings. [25] found that over 85% of students reported to be sexually active. In another study conducted among students in institutions of higher education, it was found that 61% were in a sexual relationship [14]. Moreover, our findings show that females have higher utilization rates of preventative services which is similar to prior studies [25,41]. 

## 5. Limitations

Although we endeavoured to obtain a representative sample of both genders in the college campus, the sample was overwhelmingly female. Additionally, it is possible that the study participants might have had difficulties in recalling events that took place in the past 12 months which may introduce recall bias. To address this, students were assured of anonymity of the study findings during the period of data collection. The study recruitment was limited to a single college, raising the potential for selection bias. We employed random sampling to address the bias and had a good sample size. Since we used a random methodology and the sample size was considerable, the findings can be quite generalizable to similar contexts but not the general population of youth in SA. The questionnaire responses were incomplete in some questions resulting in missing values; however, the sample size in the analysis was adjusted to account for this gap.

## 6. Conclusions

This study assessed the condom use and views on HCT of TVET college students in Limpopo Province, South Africa. The majority of the students in this study already use HCT services and know their HIV status. We found that students in this study reported a high prevalence of condom use. In fact, the college students are making efforts to ensure responsible decision making regarding their sexual health. For this reason, interventions focusing on condom use such as provision of flavoured condoms and promotion by Higher Health at TVET colleges are showing promise in achieving improved health outcomes for students. This study further reported that more female students expressed that they are comfortable taking an HIV test compared to their male counterparts, and that they are able to negotiate condom use. Using a condom the first time and knowing their partner’s HIV status are important factors to enable condom use among college students. Our findings provide useful information on HCT uptake and condom use that other colleges in the region could emulate in college HIV programming to make HCT and condoms more appealing. 

## Figures and Tables

**Table 1:** Sociodemographic, sexual behaviour and condom usage of the college students, *n* = 396.

Variables	Frequency, *n* (%)	Mean (SD)
**Sociodemographic characteristics**		
**Gender**		
Male	89 (22.8)	
Female	301 (77.2)	
**Age (years)**		22.9 (2.7)
**Areas of residence**		
Rural areas	292 (74.9)	
Urban areas	88 (22.6)	
Informal settlements	10 (2.6)	
**Level of study**		
Level 2	106 (27.9)	
Level 3	173 (45.5)	
Level 4	101 (26.6)	
**Sexual behaviour**		
**Have a sexual partner**		
Yes	339 (85.8)	
No	56 (14.2)	
**Sexually active**		
Yes	306 (81.4)	
No	70 (18.6)	
**Number of sexual partners in the past year**		
One partner	239 (63.7)	
Two partners	70 (18.7)	
More than two partners	66 (17.6)	
**Condom usage**		
**Use condom first time**		
Yes	332 (87.4)	
No	48 (12.6)	
**Use condom last time**		
Yes	225 (60.0)	
No	150 (40.0)	

**Table 2:** Reasons for testing and non-uptake of HCT, *n* = 396.

Variables	Frequency, *n* (%)
**Where HCT was conducted**	
Hospital	30 (8.6)
Clinic	265 (76.2)
Private doctor	12 (3.5)
HIV testing campaign	33 (9.5)
Other	8 (2.3)
**Reasons for testing**	
To know my status	279 (79.9)
I was pregnant	22 (6.3)
I am sexually active	9 (2.6)
Tested with my partner	14 (4.0)
I was offered a test by health provider	25 (7.2)
**Were you satisfied with HCT services**	
Satisfied	333 (96.2)
Somewhat satisfied	5 (1.5)
Not satisfied	8 (2.3)
**Reasons for HCT non-uptake**	
Not sexually active	9 (20.5)
Not at risk of contracting HIV	13 (29.6)
Not wanting to know HIV status	7 (15.9)
Not feeling comfortable testing in hospital settings	5 (11.4)
Not sure if the results will be kept confidential	4 (9.1)
Afraid of stigma	3 (6.8)
Other	3 (6.8)

**Table 3:** Views on HIV counselling and testing by gender, *n* = 396.

Variables	Females	Males	*p*-Value
**How easy is it for young people to undergo HCT**	*n* (%)	*n* (%)	
Not easy at all	190 (66.0)	58 (67.0)	0.947
Somewhat easy	21 (7.2)	7 (8.0)	
Very easy	77 (27.0)	22 (26.0)	
**HCT is important for young people**			0.854
Agree	285 (80.0)	85 (97.0)	
Disagree	6 (19.0)	1 (1.1)	
Don’t know	6 (1.6)	2 (2.2)	
**The place for HCT can motivate a person to use HTS**			0.188
Agree	242 (85.0)	66 (77.0)	
Disagree	10 (3.5)	6 (6.9)	
Don’t know	34 (11.8)	14 (16.2)	
**Have adequate information about HIV services in the facility**			0.369
Yes	155 (52.9)	39 (44.3)	
No	87 (30.0)	31(36.0)	
Unsure	51 (17.4)	18 (20.4)	
**Describe feelings about being tested for HIV**			0.002 *
Comfortable about testing for HIV	164 (54.6)	32 (36.0)	
Very scared about testing for HIV	102 (34.0)	43 (48.3)	
Not ready to test for HIV	11 (3.6)	9 (10.1)	
Intend getting tested soon	23 (7.6)	5 (5.6)	
**Know where to get free HIV testing services**			0.141
Yes	243 (81.5)	76 (86.3)	
No	42 (14.0)	6 (6.8)	
Unsure	13 (4.3)	6 (6.8)	
**Comfortable using HIV services**			0.341
Yes	236 (82.2)	68 (79.0)	
No	17 (5.9)	9 (10.4)	
Unsure	34 (11.8)	9 (10.4)	
**Worried about getting infected with HIV**			0.701
Very worried	239 (84.1)	72 (83.7)	
Somewhat worried	20 (7.0)	8 (9.3)	
Not worried	25 (8.8)	6 (6.9)	

* significant at *p* < 0.05.

**Table 4:** Logistic regression on factors associated with condom use.

Variables	Sample Distribution (%)	*p*-Value	uOR (95% CI)	aOR (95% CI)
**Gender**				
Female	89 (22.8)	0.000	2.03 (1.29, 3.20)	0.43 (0.22, 0.84)
Male	301 (77.2)			
**Sexually active**				
Yes	306 (81.4)	0.000	2.66 (1.47, 4.82)	0.37 (0.15, 0.89)
No	70 (18.6)			
**Use Condom First Time**				
Yes	332 (87.4)	0.000	3.24 (1.72, 6.10)	4.71 (2.14, 10.37)
No	48 (12.6)			
**Ever been tested for HIV**				
Yes	350 (88.4)	0.00	2.50 (1.27, 4.88)	0.89 (0.36, 2.21)
No	46 (11.6)			
**Know HIV status of partner**				
Yes	238 (60.4)	0.000	1.10 (0.77, 1.56)	2.08 (1.19, 3.65)
No	128 (32.5)			
No partner	28 (7.1)			
**Discuss HIV testing with partner**				
Likely	297 (76.0)	0.000	1.83 (1.18, 2.84)	0.81 (0.40, 1.65)
Unlikely	64 (16.4)			
Never	30 (7.7)			
**Ask partner to go for HIV testing**				
Likely	315 (81.0)	0.000	3.00 (1.75, 5.11)	0.30 (0.13, 0.67)
Unlikely	49 (12.6)			
Never	25 (6.4)			

## Data Availability

Data may be shared upon reasonable request to the authors.
